# Francis Bacon and his invisible rooms: human emotions so felt but not so easily seen

**DOI:** 10.1080/17571472.2017.1325013

**Published:** 2017-05-11

**Authors:** Francesco Carelli

**Affiliations:** Professor of Family Medicine, Milan, Italy

*Francis Bacon: Invisible Rooms* (Tate Liverpool 18 May – 18 September 2016) is the first dedicated exhibition to survey an underexplored yet significant element of Bacon’s work, an artist internationally regarded as Britain’s greatest painter of the last century and the biggest one after Turner. This exhibition helps in the re-assessment of his works in the light of the new research that has emerged since the revelation of his studio and its contents following the artist’s death.

Many of his main works feature an architectural, ghost-like framing device around his subjects. He used a cubic or elliptic cage around the figures depicted to create his dramatic compositions. These imaginary chambers are a way to emphasise the isolation of there presented figures so bringing attention to their psychological condition; just placing the sitters in ‘invisible rooms’ guides to focus attention towards the complex human emotions that are felt but can’t be seen [1].

*Francis Bacon: Invisible Rooms* traces the development of this architectural structure throughout his career. First indications of room-spaces were in early works including *Crucifixion* 1933 (Murderme Collection) and *Three Studies for Figures at the Base of a Crucifixion* c. 1944 (Tate). The 1950s saw further work on this theme, including *Man in Blue IV* 1954 (Mumok, Austria) and *Chimpanzee* 1955 (Staatsgalerie Stuttgart) which then led through to the 1980s, *Untitled (Kneeling Figure)* c. 1982 (Private Collection).

This motif was progressively developed from initial experiments on paper till theatrical spaces, described by French philosopher Gilles Deleuze, *Francis Bacon: The Logic of Sensation*, interpreted as one of the defining forces of his work.

His paintings of the 1940s bore witness to the shattered psychology of the time and shot him to a prominence that hardly diminished over the next fifty years. He captured sexuality, violence and isolation in his unflinching depictions of the anxieties of the modern condition.

Bacon’s philosophy underlines the fact that man is simply another animal in a godless world, subject to the same natural urges of violence, lust and fear that are physically evident in the body. As a consequence, Bacon’s output was dominated by the human body. His way of portraying body was unique in the history of painting, usually in isolation, at moments of extreme tension or even pain, distorted like figures from a fantastical nightmare, indicating the isolation of human life without God just as an animal. He reveals the frailty of the human figure and the scream or cry that expresses repressed and violent anxieties.

In one painting *Study for a portrait (1952)* we see the distorted human figure where we recognise the famous crying nurse on the steps depicted in Eisenstein’s movie ‘Potemkin battleship’ with broken glasses indicating the human shattered vision on a number of levels. Bacon said ‘I like Freud very much because I like the way of explaining things between conscious and unconscious’. Among his works, we see a sense of dread pervading the brutality of everyday life in which he feels humans as pathetically isolated. In contrast Bacon once said of Warhol ‘General speaking, Warhol had good subjects, he knew how to choose them very well; but his problem was that what he was doing, was alive realism, and in the end it didn’t lead to anything very interesting’ [2].

In summary, he made a daily confrontation with mortality, the inevitability and constant presence of death. Bacon has expressed with paint how human violence and sense of death had reached an apex that he tried to translate a very personal way.

## Disclosure statement

No potential conflict of interest was reported by the author.
